# High levels of interferon-gamma (IFNγ) in macrophage activation syndrome (MAS) and CXCL9 levels as a biomarker for IFNγ production in MAS

**DOI:** 10.1186/1546-0096-13-S1-O84

**Published:** 2015-09-28

**Authors:** C Bracaglia, D Pires Marafon, I Caiello, K de Graaf, F Guilhot, W Ferlin, S Davì, G Schulert, A Ravelli, A Grom, R Nelson, C de Min, F De Benedetti

**Affiliations:** 1 Division of Rheumatology Ospedale Pediatrico Bambino Gesù, Department of Pediatric Medicine, Rome, Italy; 2 Novimmune SA, Plan-les-Ouates, Geneve, Switzerland; 3 University of Genoa, Istituto Giannina Gaslini, Genoa, Italy; 4 Division of Pediatric Rheumatology, Cincinnati Children's Hospital Medical Center, University of Cincinnati College of Medicine, Cincinnati, OH, USA

## Background

A vast body of evidence in animal models points to a pivotal pathogenic role of IFNγ in primary hemophagocytic lymphohistiocytoses (HLH). High levels of IFNγ are also found in humans with HLH.

## Objectives

Given the similarities between primary and secondary HLH (sec-HLH), including MAS, we measured levels of IFNγ, IFNγ-related chemokines (CXCL9, CXCL10, CXLC11), and IL-6 in patients with sec-HLH, and in patients with systemic Juvenile Idiopathic Arthritis (sJIA) with or without MAS at sampling and evaluated their relation to disease activity. In addition, we evaluated the correlation between serum levels of IFNγ and of the three IFNγ related chemokines with themselves and with laboratory parameters of disease activity in patients with active MAS.

## Methods

We measured circulating levels of IFNγ, CXCL9, CXCL10, CXCL11 and IL-6 in patients with sJIA (n=54) of whom 20 had MAS at time of sampling using the Luminex multiplexing assay.

## Results

Levels of IFNγ and of IFNγ-related chemokines (median pg/ml(IQR)) were markedly elevated in active MAS and active sec-HLH, with no significant differences between active sec-HLH (IFNγ 34.7 (23.9-170.1); CXCL9 33598 (3083-127687); CXCL10 4420 (799.7-8226); CXCL11 1327 (189-2000)) and active MAS (IFNγ 15.4 (5.1-52.6); CXCL9 13392 (2163-35452); CXCL10 1612 (424.8-4309); CXCL11 564.8 (197.5-1007)). Levels in active sJIA without MAS at sampling were lower (all p values 2=0.47; p=0.001), to a lesser extent of CXCL10 (r=0.53; r^2^=0.28; p=0.015), and not of CXCL11 (r=-0.04;p=0.886). In active MAS ferritin, neutrophils, platelets, alanine aminotransferase and lactate dehydrogenase were significantly correlated with IFNγ and CXCL9, and to a lesser extent with CXCL10 and CXCL11; no correlation with IL-6 levels was found. In patients with active sJIA without MAS there was no significant correlation between laboratory parameters and cytokine levels (Table 1).

**Table 1:** Correlation of laboratory parameters of disease activity with IFN-g, CXCL9, CXCL10, CXCL11 and IL-6 in patients with MAS and in patients with active sJIA

	Macrophage Activation Syndrome	IFNg		CXCL9		CXCL10		CXCL11		IL-6	
		r*	*p*	r*	*p*	r*	*p*	r*	*p*	r*	*p*

Ferritin	8000 (3158 - 13174) [1]	0.57	0.014	0.49	0.041	0.66	0.002	0.62	0.023	0.17	>0.1

N	6.9 (3.4 - 13.9) [1]	-0.64	0.005	-0.61	0.010	-0.37	>0.1	-0.08	>0.1	0.09	>0.1

PLT	197 (114 - 392) [1]	-0.53	0.017	-0.52	0.022	-0.58	0.008	-0.22	>0.1	-0.02	>0.1

ALT	46 (18 - 164) [1]	0.49	0.045	0.49	0.044	0.51	0.038	0.06	>0.1	-0.44	0.080

LDH	1152 (722 - 2135) [1]	0.45	0.095	0.62	0.013	0.64	0.001	0.64	0.048	0.08	>0.1

	Sistemic Juvenile Idiopathic Arthritis	r*	*p*	r*	*p*	r*	*p*	r*	*P*	r*	*p*

Ferritin	214 (37 - 1669) [1]	-0.27	>0.1	0.28	>0.1	0.27	>0.1	0.29	>0.1	-0.12	>0.1

N	8.4 (5.2 - 14.5) [1]	0.30	>0.1	0.40	0.061	0.32	>0.1	0.40	0.067	0.28	>0.1

PLT	444 (353 - 544) [1]	0.21	>0.1	-0.14	>0.1	-0.13	>0.1	0.27	>0.1	0.35	0.064

ALT	16 (11 - 24) [1]	0.29	>0.1	0.42	0.049	0.50	0.011	0.44	0.039	0.04	>0.1

LDH	506 (455 - 851) [1]	0.07	>0.1	0.49	>0.1	0	>0.1	0.26	>0.1	0	>0.1


N=neutrophil count; PLT=platelet count; ALT=alanine aminotransferase; LDH=lactate dehydrogenase; [1]= Median (IQR); r*= Spearman r.

## Conclusions

IFNγ, and IFNγ-related chemokines, levels were increased in patients with MAS compared to patients with active sJIA without MAS. The high levels of IFNγ and of CXCL9 present in patients with active MAS were significantly correlated with laboratory parameters of disease severity. In patients with active MAS IFNγ and CXCL9 are tightly correlated. Since CXCL9 has been shown to be induced only by IFNγ and not by other interferons [1], our findings support the conclusion that CXCL9 is a potential biomarker of IFNγ production in MAS.
